# The Ethanolic Stem-Bark Extract of* Antrocaryon micraster* Inhibits Carrageenan-Induced Pleurisy and Pedal Oedema in Murine Models of Inflammation

**DOI:** 10.1155/2017/6859230

**Published:** 2017-07-17

**Authors:** Leslie B. Essel, David D. Obiri, Newman Osafo, Aaron O. Antwi, Babatunde M. Duduyemi

**Affiliations:** ^1^Department of Pharmacology, Faculty of Pharmacy and Pharmaceutical Sciences, College of Health Sciences, Kwame Nkrumah University of Science & Technology (KNUST), Kumasi, Ghana; ^2^Department of Pathology, School of Medical Sciences, College of Health Sciences, Kwame Nkrumah University of Science & Technology (KNUST), Kumasi, Ghana

## Abstract

We investigated the antioxidant and anti-inflammatory effects of a 70% v/v ethanol extract of the stem bark of* Antrocaryon micraster* on murine models of carrageenan-induced pleurisy and paw oedema. Rat pleural fluid was analysed for volume, protein content, and leucocytes, while lung histology was assessed for damage. Lung tissue homogenates were assayed for glutathione (GSH), superoxide dismutase (SOD), catalase (CAT), malondialdehyde (MDA), and myeloperoxidase (MPO). Phytochemical analysis was carried out on the stem bark. Acute toxicity studies were conducted in rats. In the pleurisy model the extract (30–300 mg/kg) significantly reduced the volume and amount of proteins and leucocytes in the exudate and also protected against lung injury. Tissue level of GSH and SOD and CAT expression were increased while MDA level and MPO activity were reduced. The peak and total oedema responses were significantly suppressed when given both preemptively and curatively in the mice paw oedema test. Saponins, alkaloids, triterpenoids, and tannins were present in the stem bark.* A. micraster* extract exhibited no apparent acute toxicity. We conclude that the ethanolic stem-bark extract of* A. micraster* has antioxidant action and exhibits significant anti-inflammatory activity through suppression of pleurisy and paw oedema induced with carrageenan.

## 1. Introduction

*Antrocaryon micraster* (Anacardiaceae) is a deciduous tree which reaches heights of 40–50 m. The tree is harvested for timber and is listed as “Vulnerable” in the International Union for the Conservation of Nature (IUCN) Red List of Threatened Species (2011) [1] and exists mostly in tropical semideciduous forests in Cameroon through Ghana to Uganda [2]. It is called “Aprokuma” in local Ghanaian communities [3] and has been noted as a medicinal plant. Medicinal plants are a rich and important source of diverse biologically active compounds which have been used as lead compounds and optimized to improve efficacy [4]. Information obtained through ethnobotanical interviews in local Ghanaian communities reveal that the stem bark, root, and leaves are boiled with water to treat malaria [5] and also rubbed on the body to treat chicken pox when it is ground with seeds of* Xylopia aethiopica* and* Aframomum melegueta* [3]. The fruits or stem bark prepared in a poultice are also taken or applied, respectively, to relieve pain and arthritic joints [6], an indication of its anti-inflammatory effect.

Inflammation is a complex process which represents a tissue's response to local injury and may result from physical and chemical insult as well as invasion by microorganisms [7] and is characterized by the classical features of oedema, redness, heat, pain, and loss of function [8]. A series of responses known as the acute inflammatory response is mounted which then isolates the site of injury or infection and/or eradicates the offending agent ultimately leading to tissue repair (sometimes with scaring) or tissue death [9, 10]. The inflammatory process is a defensive process and functions primarily to protect the tissue from further damage [11]. However, if the process is not properly regulated, or the offending agent persists, the intended protective process tends to be destructive: the underlying cause of inflammatory diseases which require pharmacological intervention [12].

Drug groups such as steroidal and nonsteroidal anti-inflammatory drugs and biologics have been developed for the management of inflammatory conditions. However, use of these drugs is limited by high costs, long duration of therapy, and adverse effects [13, 14]. Consequently, there is a renewed interest in medicinal plant research to identify alternate agents which may be cheaper and have less or no adverse effects [15].

Unfortunately, there is no report in literature to support the folklore use of any part of* A. micraster *plant in inflammatory disorders. This study therefore seeks to evaluate the aqueous ethanol extract of the stem bark of* A*.* micraster* on acute inflammation and its antioxidant properties in murine models with the view of finding a justification for its use in the management of the inflammatory conditions.

## 2. Materials

### 2.1. Plant Material

Stem bark of* Antrocaryon micraster* was harvested from Kwahu-Asakraka, Ghana (6°37′37.2072′′ N; 0°41′23.0352′′ W), in February 2015. The plant material was identified and authenticated by Professor Abraham Yeboah Mensah and a voucher specimen deposited in the herbarium of the Department of Pharmacognosy, Faculty of Pharmacy & Pharmaceutical Sciences, KNUST, Kumasi. The plant material was air-dried for 2 weeks, pulverized, and cold macerated with ethanol (70% v/v) for 72 h. The filtrate was concentrated under pressure and oven-dried at a constant temperature of 50°C for 24 h. A dark brown paste representing a yield of 7.46% w/w was obtained. When required the paste was reconstituted in normal saline (0.9% w/v NaCl) and hereby referred to as* A*.* micraster* extract (AME) in the pharmacological investigations.

### 2.2. Animals

Sprague Dawley rats (200–250 g) and Imprint Control Region (ICR) mice (20–25 g) were obtained from the Animal House, Department of Pharmacology, KNUST. Animals were housed under hygienic conditions in metal cages with wood shavings as bedding material and maintained at a 12 h light-dark cycle. The animals had free access to standard chow and clean water. All animals were humanely handled throughout the experimental period in accordance with Animal Welfare Regulations (USDA 1985; US Code, 42 USC § 289d) and the Public Health Service Policy on Humane Care and Use of Laboratory Animals (PHS 2002). All experiments were approved and clearance for commencement of the experiments was given by the Ethics Committee of the Department of Pharmacology, KNUST. Animals were humanely euthanised at the end of each experiment.

### 2.3. Drugs and Chemicals

Drugs and chemicals were obtained from the following sources; *λ*-carrageenan, Triton X-100, 5,5-dithio* bis*-2-nitrobenzoic acid (DTNB) [Sigma-Aldrich Inc., St. Louis, MO, USA]; trichloroacetic acid (TCA), thiobarbituric acid (TBA), potassium dichromate, formic acid sodium bicarbonate, chloroform, and disodium hydrogen phosphate [BDH, Poole, England]; Tris(hydroxymethyl)aminomethane and ethylenediaminetetraacetic acid (EDTA) [Techno Pharmchem Haryana, India]; diclofenac sodium [Troge, Hamburg, Germany]; sodium dihydrogen orthophosphate monohydrate [Hopkins & Williams Ltd., Swansea, Wales]; formalin, acetic acid, and analytical grade glacial acetic acid [VWR Chemicals, France]; ethanol and hydrogen peroxide [Bells Sons & Co. Ltd., Southport, England]; Complete Protease Inhibitor Cocktail Tablet, EDTA-free [Santa Cruz Biotechnology, Dallas, TX, USA]; glycerol and diethyl ether [Surechem Products Ltd., England].

## 3. Methods

### 3.1. Carrageenan-Induced Pleurisy in Sprague Dawley Rats

A method described earlier by Saleh et al. was employed [16]. Briefly, five groups of test animals (*n* = 5) were treated with either normal saline (5 ml/kg), diclofenac (10 mg/kg), or* A*.* micraster* extract (30, 100 and 300 mg/kg) daily for 3 days. Under light ether anaesthesia, pleurisy was induced in the test animals by an injection of 100 *μ*l sterile carrageenan suspension (1% w/v in normal saline) into the right pleural space 1 h after last drug treatment. The naïve control group (*n* = 5) received intrapleural injection of 100 *μ*l sterile normal saline only. After 4 h, rats were sacrificed with an overdose of diethyl ether, their thoraxes carefully opened, and the pleural cavities rinsed with 2 ml EDTA (1% w/v in normal saline). The exudate and rinse solutions were removed by aspiration and blood contaminated recovered fluid was discarded. The exudate was subjected to the following tests.

#### 3.1.1. Exudate Volume and Total Protein Content

The volume of exudate that leaked into the pleural cavity was calculated as the difference in the volumes of rinse and recovered fluids. Protein content was determined with an automated Clinical Analyser (Flexor Junior, Vital Scientific B.V., Netherlands).

#### 3.1.2. Leucocyte Cell Count

Total and differential count of leucocytes in the exudate were done with an automated analyser (Sysmex KX-21N, Sysmex America Inc., Illinois, USA).

#### 3.1.3. Histopathology of Lung Tissue

Lung tissues were carefully removed from the rats and fixed in formalin (10%). Tissues were serially dehydrated in increasing concentrations of ethanol, cleared in xylene in a TP 1020 Tissue processor (Leica Biosystems, Wetzlar, Germany), and embedded in paraffin using a Leica EG 1160 Embedding machine (Leica Biosystems, Wetzlar, Germany). Transverse sections of 5 *μ*m were cut with a Leica RM 2125 Microtome (Leica Biosystems, Wetzlar, Germany), deparaffinized, and hydrated to distilled water and stained with haematoxylin and eosin (H&E). The slides were viewed under a digital light microscope (DM 750, Leica Microsystems, Wetzlar, Germany) fitted with a digital camera (ICC 50 HD, Leica Microsystems, Wetzlar, Germany).

### 3.2. Biochemical Assays for Oxidative Stress Markers

Sprague Dawley rats were randomized into 6 groups (*n* = 5) and either untreated or treated, respectively, with normal saline (5 ml/kg), diclofenac (10 mg/kg), or* A*.* micraster* extract (30, 100, or 300 mg/kg)* p.o.* daily for 3 days. One hour after the last treatment, pleurisy was induced with carrageenan as earlier described in test animals with the naïve control group receiving intrapleural injection of 100 *μ*l sterile normal saline only. After 4 h, the animals were sacrificed and lung tissues removed, washed with PBS (pH 6.0), homogenized in ice-cold buffer [Triton X-100 (1%), protease inhibitor cocktail, Tris HCl (150 mM), NaCl (150 mM), and glycerol (10%), pH 7.4] to obtain a 10% w/v homogenate, and centrifuged at 5200 ×g for 20 min. The protein content in the supernatant was quantified using the Bradford method. The remaining samples were stored at −80°C and when needed aliquoted in triplicate and subjected to biochemical assays using the Synergy H1 Hybrid Multi-Mode Microplate Reader (BioTek Technologies, Winooski, VT, USA) for the following oxidative stress markers.

#### 3.2.1. Reduced Glutathione (GSH)

GSH levels were determined by a method earlier described by Ellman [17]. Briefly, 100 *μ*l aliquot of tissue extract was mixed with 2.4 ml EDTA (0.02 M) at 4°C for 10 min. To this 2 ml distilled water and 500 *μ*l TCA (50% w/v) were added and centrifuged at 1300 ×g for 5 min. To 1 ml of the supernatant, 50 *μ*l 5,5′-dithio-*bis-2-*nitro benzoic acid, DTNB (10 mM), and 2 ml Tris buffer (0.4 M, pH 8.9) were added. Absorbance was read within 5 min of DTNB addition at 412 nm against a blank (reagents only). The final sulphydryl concentration was extrapolated from a standard curve.

#### 3.2.2. Superoxide Dismutase (SOD) Activity

SOD activity was estimated with a modified method as earlier described by Misra and Fridovich [18]. Briefly, 500 *μ*l tissue supernatant was added to 150 *μ*l ice-cold chloroform and 750 *μ*l ethanol (96% v/v), vortexed for 1 min, and then centrifuged at 600 ×g for 20 min. To 500 *μ*l portion of the supernatant, 500 *μ*l EDTA (0.6 mM) and 1 ml carbonate bicarbonate buffer (0.1 M, pH 10.2) were added. The reaction was initiated by the addition of 50 *μ*l adrenaline (1.3 mM). Absorbance was measured at 480 nm against a blank. Activity of SOD, measured as the quantity of the enzyme required to inhibit the autooxidation of adrenaline, was then computed. SOD activity was expressed in units per mg protein, where 1 unit of enzyme activity is the quantity of enzyme required to prevent the autooxidation of adrenaline at 25°C.

#### 3.2.3. Catalase (CAT) Activity

The method described by Sinha with slight modifications was used [19]. Briefly, to a 100 *μ*l aliquot of tissue supernatant, 1 ml phosphate buffer (0.01 M, pH 7.0) and 400 *μ*l H_2_O_2_ (1.18 M) were added and the mixture was incubated at room temp for 5 min. The reaction was halted by adding 2 ml of a 3 : 1 mixture of glacial acetic acid and dichromate (5%). Absorbance was measured at 620 nm. One unit of catalase activity, defined as the amount of enzyme that degrades 1 mmol H_2_O_2_ per min at 25°C and pH 7.0, was expressed in terms of its molar extinction coefficient, 39.4 M^−1^ cm^−1^.

#### 3.2.4. Myeloperoxidase (MPO) Activity

MPO activity was determined by means of a modified* o*-dianisidine method described by Şenoğlu et al. [20]. Briefly, 150 *μ*l of the mixture consisting of 5 ml of freshly prepared 0.02 M* o*-dianisidine in deionized water, 3 ml phosphate buffer (0.1 M, pH 6.0), and 3 ml H_2_O_2_ (0.01 M) in a final volume of 30 ml was pipetted into 96-well plates in triplicate. To this, 10 *μ*l of tissue extract supernatant was added and the change in absorbance read immediately at 460 nm every min for 10 min. One unit of MPO increases absorbance by 0.001/min.

#### 3.2.5. Lipid Peroxidation and Malondialdehyde (MDA)

MDA was measured as a product of lipid peroxidation by the method of Heath and Packer [21]. Briefly, 1 ml of tissue extract was added to a 3 ml mixture of trichloroacetic acid (TCA) (20%) and thiobarbituric acid (TBA) (0.5%), heated at 95°C for 30 min and immediately cooled and centrifuged at 3600 ×g for 10 min. 200 *μ*l aliquots of supernatant was pipetted into 96-well plates and absorbance read at both 532 nm and 600 nm, respectively, to correct for nonspecific absorbance. MDA concentration (nmol/mg protein) was calculated with its molar extinction coefficient of 1.56  × 10^−5 ^M^−1 ^cm^−1^.

### 3.3. Carrageenan-Induced Paw Oedema in ICR Mice

Following the method described earlier by Winter et al. pedal oedema was induced in mice [22]. In brief, mice were injected with 50 *μ*l sterile carrageenan suspension (1% w/v) into the subplantar tissue of the right hind paw. Oedema was monitored at 1 h intervals for 6 h with an electronic calliper (model Z22855, Milomex Ltd., Bedfordshire, UK). Drug effects were evaluated by comparing the peak (maximal) and total oedema responses attained during 6 h in drug-treated groups with the corresponding values attained in saline-treated inflamed control groups. Total oedema induced during the 6 h was determined as the area under the time course curves, AUC, and used to compute the percent inhibition of the total oedema for each treatment.

In the prophylactic protocol, drug-vehicle (saline, 5 ml/kg), diclofenac (10 mg/kg), or* A*.* micraster* extract (30, 100, and 300 mg/kg), was given orally 1 h prior to the induction of the oedema while in the curative protocol treatment was 1 h after oedema induction.

### 3.4. Phytochemical Screening

The powdered plant material was subjected to phytochemical screening employing methods previously described by Sofowora [23] and Evans [24], respectively.

### 3.5. Acute Toxicity

Sprague Dawley rats were randomized into six groups (*n* = 5), placed in observation chambers, and fasted overnight but with access to water ad libitum. The animals were treated orally with either normal saline (5 ml/kg) or* A*.* micraster *extract (300, 600, 900, 1200, and 3000 mg/kg), respectively. Animals were observed at 0, 15, 30, 60, 120, and 180 min and 24 h and then daily for 14 days (delayed toxicity) for specific behaviours associated with autonomic and CNS and neurotoxicity as earlier described by Irwin [25].

### 3.6. Statistics

Data are presented as mean ± standard error of mean (SEM). Statistical differences between treatment groups were determined by one-way analysis of variance (ANOVA) complemented with post hoc testing using Dunnett's correction. Analyses were carried out with GraphPad for Windows version 6.01 (GraphPad Software Inc., San Diego, CA, USA). *p* values < 0.05 were considered statistically significant.

## 4. Results

### 4.1. Effect of* Antrocaryon micraster* Extract on Carrageenan-Induced Pleurisy

#### 4.1.1. Exudate Volume and Total Protein

**Figure 1:**
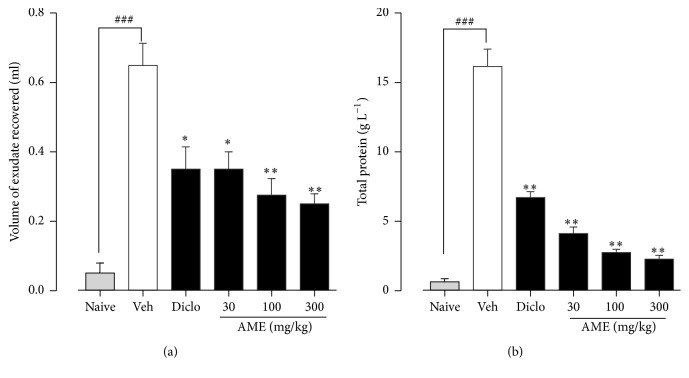
Effect of* Antrocaryon micraster* on the volume of exudate and total protein in carrageenan-induced pleurisy in rats. Sprague Dawley rats (200–250 g) were untreated or treated with either normal saline (5 ml/kg), diclofenac (10 mg/kg), or AME (30–300 mg/kg) p.o. Pleurisy was induced as described in the Methods. Volumes of exudate and total proteins were measured. Data is presented as mean ± SEM (*n* = 5). ^*∗*^*p* < 0.05 and ^*∗∗*^*p* < 0.01 compared to the vehicle-treated group; ^###^*p* < 0.001 compared to naive control (one-way ANOVA followed by Dunnett's post hoc test).

Naïve animals injected with sterile saline suspension showed no signs of pleurisy and had an average recovered exudate volume of 0.05 ± 0.03 ml (Figure 1(a)). By the single intrapleural injection of the sterile carrageenan (1% w/v), test animals suffered pleurisy characterized by an accumulation of fluid containing proteins and inflammatory cells. The vehicle-treated control animals presented an average of 0.65 ± 0.07 ml of turbid fluid (Figure 1(a)). Treatment of the test groups with* A*.* micraster* extract (30–300 mg/kg) significantly (*p* < 0.01) reduced the volume of exudate by 46.15 ± 5.04%, 57.69 ± 4.82%, and 61.54 ± 2.91%, respectively whereas diclofenac reduced the volume of exudate by 46.15 ± 6.50% (Figure 1(a)).

The mean total protein content in the exudates from the naïve control animals was 1.30 ± 0.10 g/l (Figure 1(b)). The exudate from vehicle-treated control animals contained 16.17 ± 1.93 g/l of proteins. The extract-treated animals showed statistically significant reduced amounts of proteins by 74.65 ± 2.88% and 83.15 ± 1.45% and 86.09 ± 1.71% at the doses used, respectively, compared with the vehicle-treated control. The exudate from the diclofenac-treated control group contained 6.70 ± 0.64 g l^−1^ of proteins (Figure 1(b)).

#### 4.1.2. Leucocyte Cell Count

**Figure 2:**
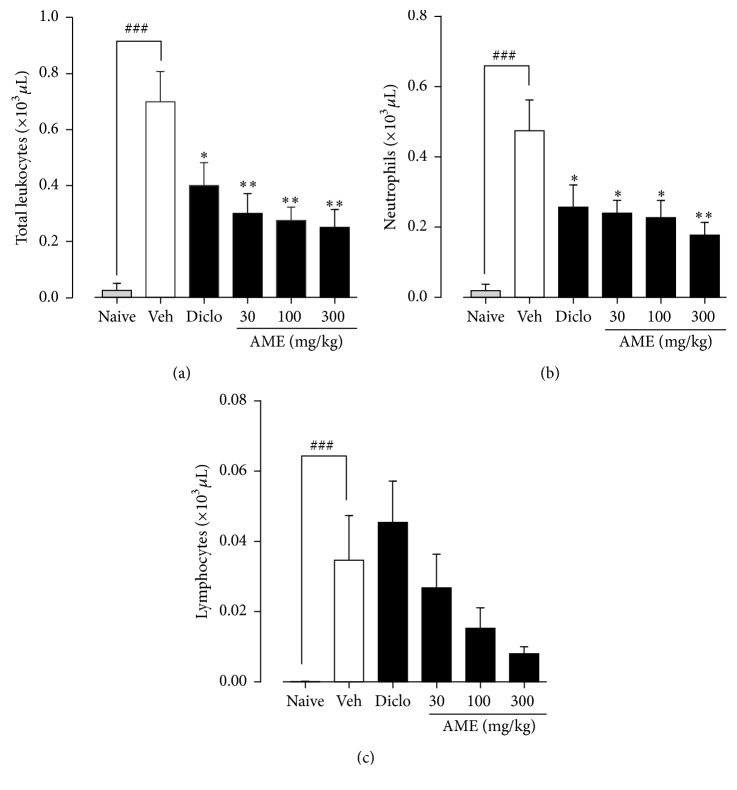
Effect of* Antrocaryon micraster* on leucocyte count in carrageenan-induced pleurisy in rats. Sprague Dawley rats (200–250 g) were untreated or treated with either normal saline (5 ml/kg), diclofenac (10 mg/kg), or AME (30–300 mg/kg) p.o. Pleurisy was induced as described in the Methods. The total and differential leucocyte count were conducted. Data is presented as mean ± SEM (*n* = 5). ^*∗*^*p* < 0.05 and ^*∗∗*^*p* < 0.01 compared to the vehicle-treated group; ^###^*p* < 0.001 compared to naive control (one-way ANOVA followed by Dunnett's post hoc test).

Analysis of the exudates revealed a total leucocyte count of 0.03 ± 0.03  ×  10^3^ *μ*L in the naïve control rats (Figure 2(a)). The exudates of the vehicle-treated control rats presented a significantly increased total leucocyte count of 0.70 ± 0.11  ×  10^3^ *μ*L compared to the naïve control rats (Figure 2(a))*. A*.* micraster* extract at 30, 100, and 300 mg/kg significantly (*p* < 0.01) inhibited the extravasation of leucocyte into the pleural cavity by 57.14 ± 10.10%, 60.71 ± 6.84%, and 64.29 ± 9.22%, respectively, compared to the vehicle-treated control (Figure 2(a)). Similarly, diclofenac significantly suppressed the total leucocyte count to 0.40 ± 0.08  × 10^3^ *μ*L. With a differential count made, the exudates of the naïve control rats had 0.02 ± 0.02  × 10^3^ *μ*L neutrophils (Figure 2(b)) which was significantly elevated to 0.47 ± 0.09  × 10^3^ *μ*L in the vehicle-treated control. The extract at the doses used also significantly (*p* < 0.05) inhibited neutrophil infiltration into the pleural cavity by 47.84 ± 10.34%, 47.94 ± 12.56%, and 50.53 ± 7.73%, respectively, whereas diclofenac significantly suppressed the neutrophil count by 44.68 ± 12.66% (Figure 2(b)). Lymphocyte numbers in the naïve control rats were below detectable levels (Figure 2(c)). However, on induction of pleurisy there was significant extravasation of these mononuclear cells into the pleural cavity to 0.04 ± 0.01  × 10^3^ *μ*L in the vehicle-treated control (Figure 2(c)). The extract at the doses administered did not have any statistically significant inhibition on the extravasation of mononuclear cells. Lymphocyte count of 0.05 ± 0.01  ×  10^3^ *μ*L was obtained in the diclofenac-treated group (Figure 2(c)).

#### 4.1.3. Effect of* Antrocaryon micraster* Extract on Lung Histopathology

**Figure 3:**
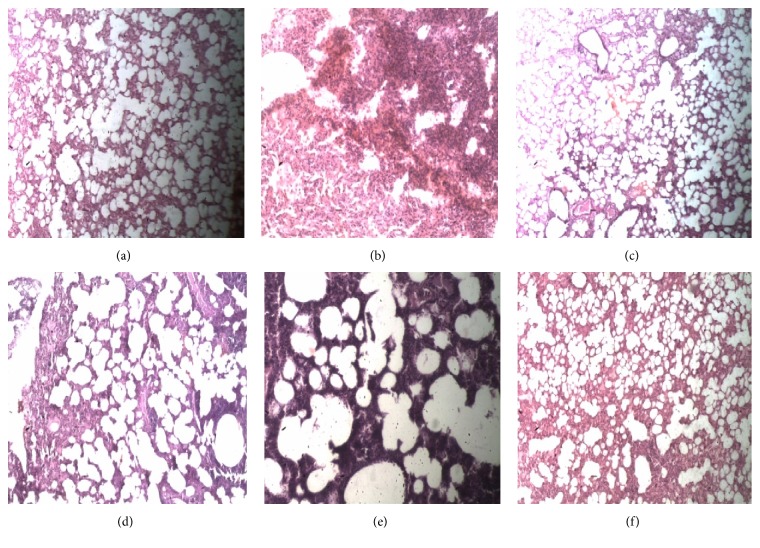
Effect of* Antrocaryon micraster* extract on histopathological changes in lung tissues of rats with carrageenan-induced pleurisy. Sprague Dawley rats (200–250 g) were untreated or treated with either normal saline (5 ml/kg), diclofenac (10 mg/kg), or AME (30–300 mg/kg)* p.o.* Pleurisy was induced as described in Methods. Lung tissues were fixed in formalin (10%), processed, and stained with H&E. Representative photomicrographs of the histology of lung tissues are shown. Naïve control (a), vehicle-treated control (b), diclofenac 10 mg/kg (c), AME 30 mg/kg (d), AME 100 mg/kg (e), and AME 300 mg/kg (f).

Histological examination of the H&E-stained lung sections of the naïve control animals showed normal architecture with no signs of damage, haemorrhage, oedema, or inflammatory cells (Figure 3(a)). In the vehicle-treated control group there was damage to lung tissues characterized by a significant distortion of the lung histological architecture, massive infiltration of inflammatory cells, oedema, and haemorrhage (Figure 3(b)).* A*.* micraster* extract when administered at 30–300 mg/kg significantly reduced the degree of lung injury by offering some protection. The histological architecture of the lungs was mostly preserved and there was a significantly reduced numbers of infiltrating cells and less oedema and haemorrhage when compared with the vehicle-treated control animals (Figures 3(d)–3(f)). Administration of diclofenac protected against lung tissue damage and reduced the oedema, haemorrhage, and the number of infiltrating cells (Figure 3(c)).

### 4.2. Biochemical Assays for Oxidative Stress Markers

#### 4.2.1. Effect of* Antrocaryon micraster* Extract on Oxidative Stress Markers

**Figure 4:**
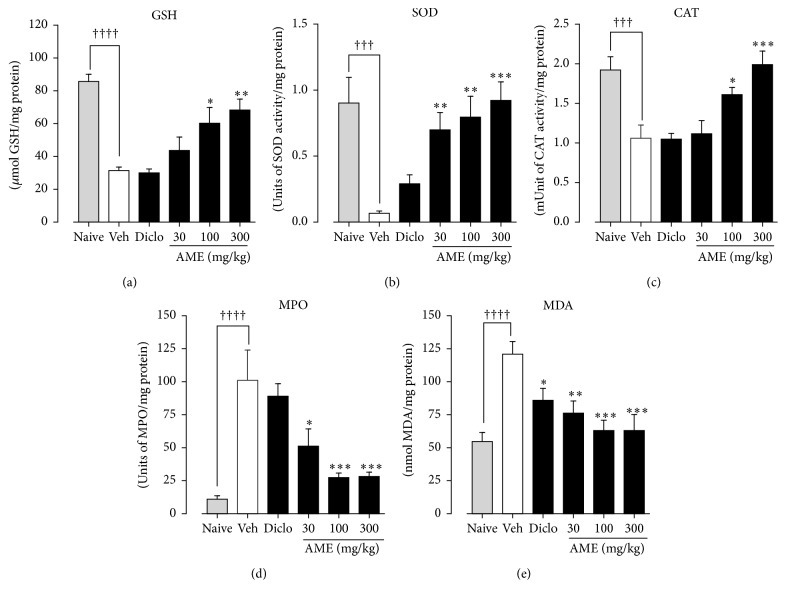
Anti-oxidant effect of* Antrocaryon micraster* extract on carrageenan-induced pleurisy in rats. Sprague Dawley rats (200–250 g) were untreated or treated with either normal saline (5 ml/kg), diclofenac (10 mg/kg), or AME (30–300 mg/kg) p.o. for 3 days. Pleurisy was induced as described in the Methods. Lung homogenates were assayed for GSH (a), SOD (b), CAT (c), MPO (d), and MDA (e). Data is presented as mean ± SEM (*n* = 5). ^*∗*^*p* < 0.05, ^*∗∗*^*p* < 0.01, and ^*∗∗∗*^*p* < 0.001 compared to vehicle-treated control; ^†††^*p* < 0.001 and ^††††^*p* < 0.0001 compared to naïve control (one-way ANOVA followed by Dunnett's post hoc test).

Carrageenan-induced oxidative stress in the test animals was evidenced by a statistically significant decrease in the measured levels of GSH and expression of SOD and CAT in the vehicle-treated control animals when compared to the naïve controls (Figures 4(a)–4(c)). The level of GSH in the naïve control group of 85.70 ± 4.45 *μ*mol/mg protein was significantly reduced in the vehicle-treated control group to 31.45 ± 2.10 *μ*mol/mg protein (Figure 4(a)).* A. micraster* extract at 30, 100, and 300 mg/kg significantly increased levels of GSH to 43.72 ± 8.15 *μ*mol/mg protein, 60.29 ± 9.60 *μ*mol/mg protein, and 68.37 ± 6.66 *μ*mol/mg protein, respectively, in comparison to the vehicle-treated control rats, while in the diclofenac-treated rats a GSH concentration of 30.08 ± 2.34 *μ*mol/mg protein was obtained (Figure 4(a)).

The expression of SOD in naïve control rats at 0.90 ± 0.20 units/mg protein was significantly reduced to 0.07 ± 0.02 units/mg protein in the vehicle-treated control group (Figure 4(b)).* A*.* micraster* extract at the stated doses significantly increased the expression of SOD to 0.70 ± 0.13 units/mg protein, 0.80 ± 0.16 units/mg protein, and 0.92 ± 0.14 units/mg protein, respectively, compared to the vehicle-treated control group while in the diclofenac-treated rats SOD concentration was 0.30 ± 0.07/mg protein (Figure 4(b)).

In the naïve control group CAT measured 1.92 ± 0.17 units/mg protein and was significantly reduced to 1.06 ± 0.17 munits/mg protein in the vehicle-treated control group (Figure 4(c)). However,* A*.* micraster* extract at the same doses significantly increased the expression of CAT to 1.12 ± 0.17 munits/mg protein, 1.61 ± 0.1 munits/mg protein, and 1.99 ± 0.17 munits/mg protein, respectively, compared to the disease vehicle-treated control. Diclofenac-treated rats presented with CAT expression of 1.05 ± 0.07 munits/mg protein (Figure 4(c)).

Increased expression of MPO and MDA levels was observed as marker for the induction of oxidative stress by carrageenan in the disease vehicle-treated control animals (Figures 4(d) and 4(e)) relative to the naïve rats. The disease vehicle-treated control group had a statistically significant increased MPO activity of 101.2 ± 22.58 units/mg protein relative to naïve control group of 11.01 ± 2.67 units/mg protein (Figure 4(d)).* A*.* micraster* extract significantly reduced the expression of MPO to 51.22 ± 13.00 units/mg protein, 27.42 ± 3.46 units/mg protein, and 28.83 ± 3.23 units/mg protein, respectively, relative to the vehicle-treated control group. A level of 89.06 ± 9.40/mg protein was obtained in the diclofenac-treated group (Figure 4(d)).

MDA level in naïve control group was 54.77 ± 6.76 nmol/mg protein with the vehicle-treated control group significantly increasing it to 121.0 ± 9.59 nmol/mg protein (Figure 4(e)). Similar statistically significant reductions in MDA to 76.28 ± 9.17 nmol/mg protein, 63.01 ± 7.87 nmol/mg protein, and 63.07 ± 12.05 nmol/mg protein, respectively, compared to the vehicle control group were obtained at the three respective doses of the extract. Relative to the disease vehicle-treated control group, diclofenac decreased the concentration of MDA to 85.93 ± 9.17 nmol/mg protein (Figure 4(e)).

### 4.3. The Effect of* Antrocaryon micraster* Extract on Carrageenan-Induced Paw Oedema

**Figure 5:**
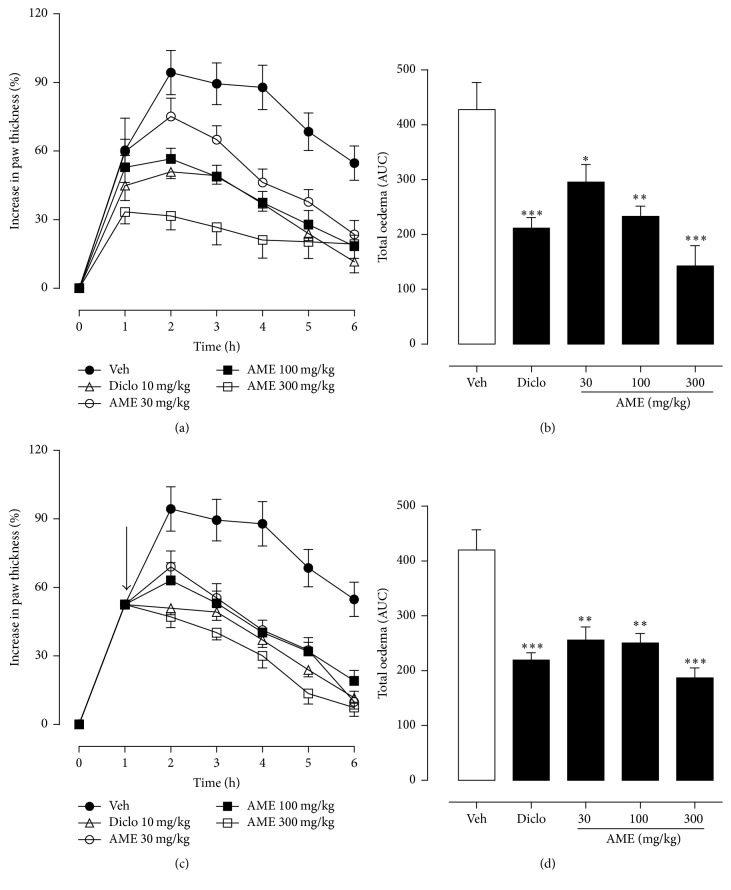
Effect of* Antrocaryon micraster* extract on carrageenan-induced paw oedema in mice. Oedema was induced and monitored as described in the Methods. Percentage increase in paw thickness (a and c) and total oedema induced during the 6 h was calculated as area under the time course curves, AUC (b and d). Drug vehicle, AME 30–300 mg/kg, and diclofenac 100 mg/kg were given in the prophylactic (top panel) or curative protocol (bottom panel). Data is presented as mean ± SEM (*n* = 5). ^*∗*^*p* < 0.05, ^*∗∗*^*p* < 0.01, and ^*∗∗∗*^*p* < 0.001 compared to vehicle-treated group (one-way ANOVA followed by Dunnett's post hoc test). Arrow indicates point of extract administration in the curative protocol.

Injection of the carrageenan suspension into the right hind paws of the test mice produced a time-dependent increase in paw thickness which peaked at the 2 h and was significantly and dose-dependently reduced by the prophylactic administration of* A*.* micraster* extract. The maximal oedema response was suppressed by 20.34 ± 1.70%, 40.48 ± 2.22%, and 66.43 ± 3.54%, respectively, at 30, 100, and 300 mg/kg of AME compared with the vehicle-treated control (Figure 5(a)). Total oedema over the 6 h was also suppressed by 30.86 ± 7.38%, 45.55 ± 4.40%, and 66.68 ± 8.60%, respectively, compared with the vehicle-treated control (Figure 5(b)). At the same doses in the curative protocol,* A*.* micraster* extract significantly (*p* < 0.05) reduced maximal oedema response by 26.77 ± 2.78%, 33.13 ± 1.95%, and 50.11 ± 4.99%, respectively (Figure 5(c)), and inhibited total oedema response by 39.11 ± 5.69%, 40.37 ± 4.15%, and 55.45 ± 4.25%, respectively, compared to the vehicle-treated control (Figure 5(d)). As was expected, diclofenac inhibited both the maximal and total oedema responses by 50.85 ± 2.87% and 50.52 ± 3.82%, respectively, in the preventive protocol and 50.73 ± 4.22% and 50.13 ± 2.48%, respectively, in the curative protocol (Figures 5(a)–5(d)).

### 4.4. Phytochemical Screening

Phytochemical analysis of the ethanolic stem bark of* A*.* micraster* tested positive for the presence of saponins, alkaloids, triterpenoids, and both hydrolysable and condensed tannins.

### 4.5. Acute Toxicity

There was no death recorded within 24 h and up to 14 days of observation. The doses of* A*.* micraster *again did not cause any apparent changes associated with autonomic or central nervous system or neurotoxicity. The LD_50_ for the* A*.* micraster* extract is greater than 3000 mg/kg.

## 5. Discussion

In this study, the effect of* Antrocaryon micraster* extract on acute inflammation was evaluated in two related experiments in which carrageenan was used to induce pleurisy and pedal oedema, respectively, in rats and mice. We could show that, in the pleurisy model, the extract exhibited significant inhibitory effects on cellular infiltration, as well as fluid and protein leakage into the pleural cavity. Again, pretreatment with the extract protected against lung tissue damage through a significant reduction in cellular infiltration, oedema, and haemorrhage to the lung tissues. Tissue levels of the oxidative stress markers GSH and expression of SOD and CAT were increased while levels of MDA and MPO were reduced. In the pedal oedema model, the maximal and total oedema responses, respectively, in mice were significantly suppressed even when the extract was given prophylactically or curatively. Our results demonstrate that the effect of the extract on the paw oedema study is in consonance with that obtained in the pleurisy study and further validates the suppressive effect of* A*.* micraster* extract on acute inflammation.

The carrageenan-induced paw oedema model is well-characterized, standardized [26, 27], and employed largely in the study of mechanisms involved in the inflammatory response and predominantly leucocyte-driven reactions and for screening potential anti-inflammatory agents that have effects on such reactions [28, 29]. The development of oedema after the carrageenan injection happens in two phases. In the early phase, autocoids such as serotonin and histamine are implicated and this phase lasts for about an hour. Prostaglandins and the induction of cyclooxygenase-2 (COX-2) predominate in the late phase which stretches from 1 to 6 h [30, 31]. Prostaglandins act together with vasoactive substances to increase vascular permeability and enhance the accumulation of fluids in the tissues [32]. Again, in response to the injected phlogistic agent, leucocytes become activated. There is incomplete reduction of oxygen within the cell leading to the production of reactive oxygen species (ROS) also called free radicals notably superoxide anion (O_2_^–^) and hydroxyl radical (HO^−^) [33] and the peroxidation of lipid membranes. When large amounts of ROS are generated by leucocytes during respiratory burst it leads to oxidative stress and tissue injury and remodelling [34, 35] through the modulation of the release of mediators and regulate the expression of adhesion molecules [36, 37]. A battery of antioxidant molecules such as GSH, SOD, and CAT act on the free radicals when generated. However, oxidative stress occurs when these defence mechanisms are overwhelmed or depleted. Oxidative stress has been implicated in the aetiology of many inflammatory disease models including pleurisy induced by carrageenan administration. An extract that increases these antioxidants tends to protect against damage to tissues. Our results show* A*.* micraster* extract inhibited the effect of oxidative stress by increasing the levels of GSH and expression of SOD and CAT while reducing MPO activity and MDA level. Increased GSH and SOD and CAT have been established as a result of the inhibition of their depletion and/or an augmentation in their production, in response to the induced oxidative stress [38, 39]. The tissue levels of MDA and activity of MPO correlate with the number of infiltrating cells in the tissue [40]. Analysis of the pleural fluid obtained from the extract-treated test animals showed reduced numbers of leucocytes, which explains the reduced MPO activity and MDA levels. The administration of* A*.* micraster* extract possibly affected these parameters by inhibiting the infiltration and effect of activated leucocytes in the tissues. This was confirmed by the histopathological findings which showed reduced number of cellular infiltration as well as attenuated lung tissue damage with the extract administration. In a previous study carried out in the absence of pathology and in which daily administration was done for 28 days, diclofenac is documented to increase the activity of SOD but not CAT [41]. However, in our study, diclofenac did not have significant effects on the activity of MPO, expression of SOD and CAT, and the levels of GSH but decreased the levels of MDA. In the present study, diclofenac was administered daily at 10 mg/kg for 3 days and its effect on carrageenan-induced oxidative stress evaluated. Diclofenac reduced exudate volume and protein leakage into pleural cavity. This way, it prevented oxidative stress, but not through increasing the levels of antioxidants. Our results are consistent with previous findings that doses above 80 mg/kg of diclofenac have been used to induce oxidative stress [42, 43] even though lower doses inhibit oxidative stress. The extract demonstrated dose-dependent inhibitory effects on acute inflammation when administered both prophylactically and curatively in the paw oedema test. This is interesting because it has been shown previously that proven anti-inflammatory activity for a drug which is administered before initiation of the inflammatory response does not necessarily imply an ability to act curatively. For example, it was shown in rats that preemptive administration of cyclosporin prevented the onset of collagen-induced inflammation but treatment with the drug after the onset of disease rather exacerbated the condition [44].

In this study, the phytochemicals identified in the crude extract of* A*.* micraster* were tannins, saponins, triterpenoids, and alkaloids. It is not surprising therefore that* A*.* micraster *extract exhibited anti-inflammatory activity. In support of this finding it has been previously reported these phytochemicals possess anti-inflammatory activity in various disease models of inflammation. For example, the tannins present in* Punica granatum* exhibited anti-inflammatory activity through the inhibition of nitric oxide synthase [45], saponins derived from* Platycodon grandiflorum* inhibited carrageenan-induced inflammation in rats [46], and the triterpenoid pristimerin derived from the Celastraceae family modulates cellular and soluble immune mediators of inflammation [47]. Previously, we had also shown that the total alkaloid extract of* Picralima nitida* seeds has anti-inflammatory activity [48]. The dose-dependent suppression of carrageenan-induced mouse pedal oedema by the extract could most likely indicate that one or more of the constituent compounds potentially inhibited the acute phase of inflammatory processes though the precise mechanism of action of* A*.* micraster *extract is yet to be determined. The constituent bioactive compounds in plant extracts impact on the toxicity of the plant [49, 50]. There was no death and no apparent behavioural changes observed within 24 h and up to 14 days of observation. Thus* A*.* micraster* extract has no immediate or delayed physically apparent acute toxicity effect and has an LD50 greater than 3000 mg/kg.

Taken together, the ethanolic stem-bark extract of* A*.* micraster *has antioxidant effects and significantly inhibits carrageenan-induced inflammation in murine models.
